# Phosphorus and Compost Management Influence Maize (*Zea mays*) Productivity Under Semiarid Condition with and without Phosphate Solubilizing Bacteria

**DOI:** 10.3389/fpls.2015.01083

**Published:** 2015-12-07

**Authors:** Amanullah, Adil Khan

**Affiliations:** Department of Agronomy, Faculty of Crop Production Sciences, The University of Agriculture, PeshawarPeshawar, Pakistan

**Keywords:** maize, *Zea mays*, grain yield, PSB, compost time, phosphorus levels

## Abstract

Phosphorus (P) unavailability and lack of organic matter in the soils under semiarid climates are the two major constraints for low crop productivity. Field trial was conducted to study the effects of P levels, compost application times and seed inoculation with phosphate solubilizing bacteria (PSB) on the yield and yield components of maize (*Zea mays* L., cv. Azam). The experiment was conducted at the Agronomy Research Farm of The University of Agriculture Peshawar-Pakistan during summer 2014. The experiment was laid out in randomized complete block design with split plot arrangement using three replications. The two PSB levels [(1) inoculated seed with PSB (+) and (2) seed not inoculated with PSB (- or control)] and three compost application times (30, 15, and 0 days before sowing) combination (six treatments) were used as main plot factor, while four P levels (25, 50, 75, and 100 kg P ha^-1^) used as subplot factor. The results confirmed that compost applied at sowing time and P applied at the two higher rates (75 and 100 kg P ha^-1^) had significantly increased yield and yield components of maize under semiarid condition. Maize seed inoculated with PSB (+) had tremendously increased yield and yield components of maize over PSB-control plots (-) under semiarid condition.

## Introduction

Maize (*Zea mays* L.) is the third most important cereal crop in Pakistan after wheat and rice (Farhad et al., 2009). In Northwest Pakistan (Khyber Pakhtunkhwa province) maize ranked second after wheat in its importance (Amanullah et al., 2012). Maize average yield in Northwest Pakistan is too low as compared to the average yield of the country (Amanullah et al., 2009, 2010). During 2012, maize was cultivated on an area of 1087.3 thousand hectares with the total production of 4338.3 thousand tons and national average yield of 3990 kg ha^-1^ in the country, while in Khyber Pakhtunkhwa it was grown on about 475.3 thousand hectares with a total production of 887.8 thousand tones and average yield of 1868 kg ha^-1^ (MINFAL, 2012). The major problems under semiarid condition in Northwest Pakistan are (1) low soil moisture and (2) low soil fertility especially P unavailability (Amanullah et al., 2012). Phosphorus is very important for improving crop growth and yield (Williamson et al., 2001; Gupta, 2003; Wu et al., 2005). Unfortunately under semiarid condition plants are not able to get the required P (Gill et al., 2004; Aziz et al., 2005; Shenoy and Kalagudi, 2005) due to high soil pH (Aziz et al., 2005) and low organic matter (Amanullah et al., 2012, 2014; Iqbal et al., 2015). Deficiency of soil P is one of the important factors restricting maize growth and yield in semiarid climates (Vance et al., 2003; Gyaneshwar et al., 2006). Phosphorus adsorption under calcareous soils (Johnston, 2001; Ibrikci et al., 2005; Amanullah et al., 2009, 2010) in semiarid condition (Hao et al., 2002; Gill et al., 2004; Aziz et al., 2005) decrease availability of P for the crop plants. Investigating proper P rates for improving crop productivity is of significant importance under semiarid climates (Amanullah and Khan, 2011; Amanullah et al., 2012).

As most of the soils under semiarid climates having less organic matter (Azad and Yousaf, 1982; Hidayatullah et al., 2013; Iqbal et al., 2015). Increase in crop productivity has been observed under semiarid climates with the addition of organic materials into the soil (Hidayatullah et al., 2013; Iqbal et al., 2015). Organic matter increases soil fertility, nutrients availability and improve soil structure (Chang et al., 1993). Organic fertilizers includes farmyard manure, animal manure, compost, plant residues and bio-fertilizers (Sobulo and Babalola, 1992; Izunobi, 2002; Stefan, 2003; Khan et al., 2005; Amujoyegbe et al., 2007; Hidayatullah and Amanullah, 2015). Compost is mixture of organic residues that has been piled, moistened, and allowed to decompose in a pit or heap. The use of compost in crop production is considered very important by managing large volumes of organic wastes and improvement in crop production (Lasaridi and Stentiford, 1999). Composted organic material can be used as a source of important nutrients for sustainable crop productivity under semiarid climates (Amanullah et al., 2015). Korsaeth et al. (2002) reported that application of compost is important in sustaining farming by providing plant N-supply to crops. Application of composted feedlot manure increases maize yield similar to commercial fertilizers (Eghball and Power, 1999; Ahsok et al., 2005).

A diverse group of soil microorganisms are involved in solubilizing insoluble P complexes enabling plants to easily absorb P (Tripura et al., 2005). Many kinds of soil bacteria (*Bacillus, Pseudomonas, Rhizobium, and Enterobacter*) and fungi (*Aspergilllus and Penicillium*) have the skill to change insoluble form of P in the soil into soluble form through releasing organic acids such as formic acids, propionic acids, acetic acids, fumaeric acids, and succinic acids (Parassana et al., 2011; Walpola and Yoon, 2012). The use of beneficial microorganisms (bio-fertilizers) such as phosphate solubilizing bacteria (PSB) as inoculants with the seed increases P availability and uptake by the plants (Mousavi et al., 2004; Sharan et al., 2008; Nico et al., 2012) because the beneficial microbes produce of organic acids which reduce soil pH (Chen et al., 2006; Rodriguez et al., 2006). These acids reduce the _P_H and bring the dissolution of bound forms of phosphate (Walpola and Yoon, 2012). Beneficial microorganisms are important not only for the reduction of the quantity of chemical fertilizers and environment friendly (Hafeez et al., 2002) but also increased crop productivity (Afzal et al., 2005; Canbolat et al., 2006; Gyaneshwar et al., 2006; Yasmin and Bano, 2011).

Organic agriculture is important for the improvement crop growth, yield (Amanullah and Stewart, 2015; Amanullah et al., 2015), environmental conditions and human health (Kurtar and Ayan, 2004; Zengin, 2007). As deficiency of P and organic matter are some of the major limiting factors for crop production under semiarid condition. Application of compost and PSB could increase soil organic matter, P availability and crop productivity. Use of proper organic and inorganic fertilizer combination, is a key factor in crop production for sustainable agriculture under semiarid climates (Hidayatullah et al., 2013; Iqbal et al., 2015). Under semiarid climates, there is no published research on the interactive effects of compost application time and P levels with and without PSB. This research work was therefore designed with an objective to find out proper timing of compost application along with suitable P level with and without PSB for improving yield and yield components of maize under semiarid climates.

## Materials and Methods

### Experimental Site

Field experiment was conducted to investigate the impact of three compost (2 t ha^-1^) application times [0, 15, and 30 days before sowing (DBS)], four phosphorus levels (25, 50, 75, and 100 kg P ha^-1^), and two PSB levels [(1) inoculated seed with PSB (+) and (2) seed not inoculated with PSB (- or control)] on the yield and yield components of maize (*Zea mays* L., cv. Azam). The research was conducted at the Agronomy Research Farm of The University of Agriculture Peshawar, during summer 2014. The experimental farm is located at 34.01° N latitude, 71.35° E longitude at an altitude of 350 m above sea level. The farm Soil is silt clay loam, low in organic matter (0.87%), extractable P (5.6 mg P kg^-1^), exchangeable, alkaline (pH 8.2) and is calcareous in nature (Amanullah et al., 2010).

### Experimentation

The experiment was laid out in randomized complete block design with split plot arrangement using three replications. Combination of factor-A (compost application times) and factor-B (inoculated seed with PSB and seed not inoculated with PSB) was used as main plot factor (six treatments) and factor-C (four P levels) used as subplots factor. A sub-plot size of 4 m × 3.5 m, having five rows, 4 m long and 70 cm apart were used. The required amount (2 t ha^-1^) of commercial compost “*Higo Organic Plus*” was applied at 0, 15, and 30 DBS. The chemical analysis of *Higo Organic Plus* was earlier reported by Amanullah et al. (2015). The required P (25, 50, 75, and 100 kg P ha^-1^) was applied seedbed preparation just before sowing of the crop. The PSB was obtained from the National Agricultural Research Center Islamabad was mixed with the seed (the plots with PSB) before sowing of the crop. A uniform dose of 120 kg N ha^-1^ as urea in two equal splits was applied half at sowing and half at knee height. The local maize cultivar “*Azam*” was used as test crop in the experiment. All other agronomic practices were kept uniform and normal for all the treatments.

### Factors and Their Levels

Factor A: Compost application time (2 t ha^-1^)

(1) 30 days before sowing
(2) 15 days before sowing
(3) 00 days before sowing (at sowing)

Factor B: Phosphate solubilising bacteria (PSB)

(1) Seed not inoculated with PSB (-)
(2) Seed inoculated with PSB (+)

Factor C: Phosphorus Levels (kg P ha^-1^)

(1) 25
(2) 50
(3) 75
(4) 100

### Data Recording

Data were recorded on number of grains ear^-1,^ number of grains row^-1^, 1000 grains weight, grain yield, harvest index and shelling percentage.

### Number of Grains per Ear

Number of grains ear^-1^ was calculated on ten randomly selected ears from each subplot and then average was calculated.

### Number of Grain Rows per Ear

Data on number of grains row^-1^ of ears was calculated by counting grains rows of ten selected ears and then average was calculated.

### Thousand Grains Weight

Grains weight of randomly 1000 grains was taken from seed lot of each subplot three times and weighed with the help of electronic balance and then average weight was calculated.

### Grain and Biological Yields

Data on biological yield was recorded by harvesting three central rows in each plot, the material was sun dried for several days and weighed, and then converted into biological yield (kg ha^-1^). The ears of the three central rows were separated from the harvested material for the biological yield. The ears were threshed, cleaned and weighed and then converted into grain yield (kg ha^-1^).

Harvest index for each treatment was calculated by using the following formula:

$$Harvestindex(\%)=\frac{Grainyield}{Biologicalyield}\times 100$$

Shelling percentage for each treatment was calculated by using the following formula:

$$Shellingpercentage=\frac{Grains\;weight\;of\;10\;ears}{Totalweightof10ears}\times 100$$

### Statistical Analysis

The collected data of all parameters were subjected to analysis of variance (ANOVA) and statistically analyzed according to Steel et al. (1997) for randomized complete block design with split plot arrangement and means among different treatments were compared using least significant differences (LSD) test (*p* ≤ 0.05).

## Results and Discussion

### Maize Response to Phosphorus Levels

Phosphorus levels had significantly (*P* ≤ 0.05) affected number of grains ear^-1^ and grains row^-1^, 1000 grains weight, grain yield, harvest index and shelling percentage (**Tables 1** and **2**). Phosphorus applied at the two higher rates (75 and 100 kg P ha^-1^) had increased number of grains ear^-1^ and grains row^-1^, 1000 grains weight, grain yield, harvest index and shelling percentage. Improvement in yield and yield components of maize has been recorded earlier with application of P (Farhad et al., 2009; Balochgharayi, 2011). Hussain et al. (2006) and Amanullah et al. (2010) reported that yield and yield components of maize increased significantly with increase in P level upto 90 kg P ha^-1^. Amanullah et al. (2010) also suggested that application of 90 kg P ha^-1^ to maize crop increased grower’s income as compared to the low rates of P (90 > 60 > 30 kg P ha^-1^). In the current experiment, phosphorus applied at the two lower rates (25 and 50 kg P ha^-1^) had significantly produced less yield and yield components (**Tables 1** and **2**). Vance et al. (2003) and Gyaneshwar et al. (2006) reported decrease in yield with P deficiency in soil. Amanullah et al. (2010) reported that decrease in P level not only decreased yield and yield components of maize but also declined the income of maize growers under semiarid climates.

**Table 1:** Number of grain ear^-1^, number of grains row^-1^ and thousand grains weight of maize as affected by affected by phosphorus levels (P), compost application time (C) and phosphate solubilizing bacteria (PSB).

Phosphorus levels (kg P ha^-1^)	Grains ear^-1^	Grains row^-1^	Thousand Grains weight (g)
25	324^c^	34^c^	232.9^b^
50	347^b^	36^b^	236.2^b^
75	371^a^	39^a^	245.7^a^
100	377^a^	39^a^	247.0^a^
LSD_0.05_	10.1	0.91	3.61
**Compost (days before sowing)**			
30	343^b^	36^c^	237.3^b^
15	348^b^	37^b^	240.2^ab^
00	373^a^	39^a^	244.0^a^
LSD_0.05_	9.7	0.72	4.98
**PSB**			
Un-inoculated seeds	347^b^	36^b^	237.8^b^
Inoculated seeds	362^a^	38^a^	243.1^a^
LSD_0.05_	1.3	0.09	0.67
**Interactions**			
C × P	^∗^(**Figure 1**)	ns	ns
C × PSB	ns	^∗^(**Figure 2**)	^∗^(**Figure 4**)
P × PSB	ns	^∗^(**Figure 3**)	ns
C × P × PSB	ns	ns	ns

Means followed by different letters within each category are significantly different using LSD test (*p* ≤ 0.05). ns, non-significant at 5% level of probability; *, significant at 5% level of probability.

**Table 2:** Grain yield, harvest index and shelling percentage of maize as affected by affected by phosphorus levels (P), compost application time (C) and phosphate solubilizing bacteria (PSB).

Phosphorus levels (kg P ha^-1^)	Grain yield (kg ha^-1^)	Harvest index (%)	Shelling Percentage (%)
25	3788^c^	35.0^b^	77.0^d^
50	3976^b^	35.5^b^	78.3^c^
75	4481^a^	37.8^a^	81.4^b^
100	4521^a^	37.6^a^	82.4^a^
LSD_0.05_	73	0.87	0.79
**Compost (days before sowing)**			
30	4066^c^	36.1^a^	77.9^c^
15	4203^b^	36.5^a^	79.9^b^
00	4306^a^	36.8^a^	81.5^a^
LSD_0.05_	73	ns	1.14
**PSB**			
Un-inoculated seeds	4097^b^	36.3^a^	78.5^b^
Inoculated seeds	4286^a^	36.6^a^	81.1^a^
LSD_0.05_	10	ns	0.15
**Interactions**			
C × P	^∗^(**Figure 5**)	ns	ns
C × PSB	ns	ns	^∗^(**Figure 7**)
P × PSB	^∗^(**Figure 6**)	ns	ns
C × P × PSB	ns	ns	ns

Means followed by different letters within each category are significantly different using LSD test (*p* ≤ 0.05). ns, non-significant at 5% level of probability; *, significant at 5% level of probability.

### Maize Response to Compost Application Time

Compost application time had significant (*P* ≤ 0.05) effects number of grains ear^-1^, number of grains row^-1^, 1000 grains weight, grain yield and shelling percentage. Compost application time had no significant effect on harvest index of maize. Application of compost at sowing time increased yield and yield components of maize (**Tables 1** and **2**). The increase in the yield and yield components of maize with compost applied at sowing time may be attributed to the availability of nutrients (Biala, 2000). Application of composted feedlot manure increases maize yield similar to commercial fertilizers (Eghball and Power, 1999; Ahsok et al., 2005). Earlier, Amanullah et al. (2015) found that application of compost (2 t ha^-1^) was most beneficial in terms of higher yield and yield components of maize over control (compost not applied). Improvement in yield and yield components with application of organic matter has been reported by Fagimi and Odebode (2007), Garg and Bahla (2008), Hidayatullah et al. (2013) and Iqbal et al. (2015).

#### Maize Response to Compost and Phosphorus Interaction

**FIGURE 1:**
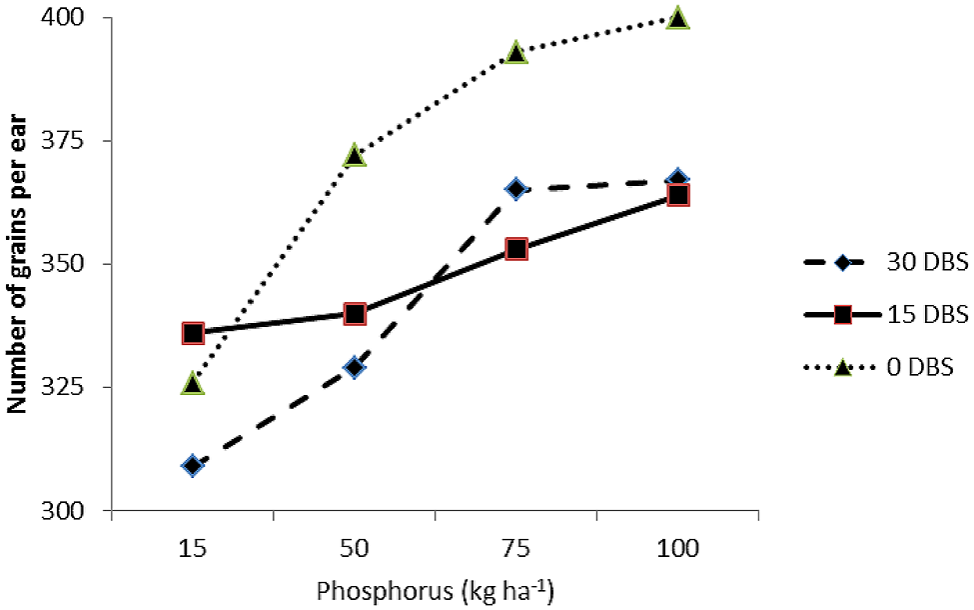
**Response of number of grains per ear of maize (*Zea mays* L.) to phosphorus levels (P) and compost application time (C) interaction (P × C)**.

**FIGURE 2:**
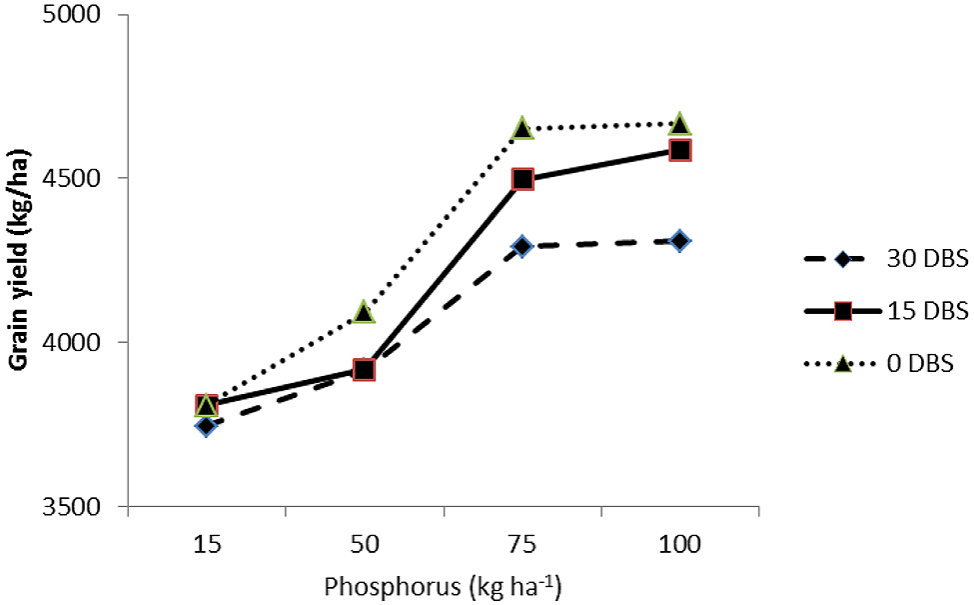
**Response of number of grain yield (kg/ha) of maize (*Zea mays* L.) to phosphorus levels (P) and compost application time (C) interaction (P × C)**.

The interaction between compost application time and P levels (C × P) had significant effect on number of grains per ear (**Figure 1**) and grain yield (**Figure 2**). **Figure 1** indicated that at the lowest P level (15 kg P ha^-1^) the three compost application time had produced almost similar number of grains per ear. At all other three levels of P (50, 75, and 100 kg P ha^-1^), application of compost at sowing time (0 DBS) had significantly produced more numbers of grains per ear as compared with 15 and 30 DBS (**Figure 1**). The C × P interaction also indicated that the two higher P levels (75 and 100 kg P ha^-1^) had produced significantly higher grain yield in maize at all three compost application times (**Figure 2**). However, the delay in compost application time was found better by producing higher grain yield (**Figure 2**). Decrease in P level and early application of compost was found to decrease maize yield. According to Amanullah et al. (2015), application of compost at the rate of 2 t ha^-1^ along with 120 kg N ha^-1^ was declared the best combination to yield and yield components of maize. Increase in yield and yield components of maize was reported earlier by Cheema et al. (2010), Zafar et al. (2011), Khan et al. (2013) and Iqbal et al. (2015) due to integrated application of organic and inorganic fertilizers under semiarid climates. The increase in yield of maize with combined application of P and compost probably may be due to the increase in P availability (Biswas, 2011).

#### Maize Response to Phosphate Solubilizing Bacteria

Significant differences were found in number grains ear^-1^ and grains row^-1^, 1000 grains weight, grain yield and shelling percentage between the plots treated with PSB (+) and without PSB (-) as shown in **Tables 1** and **2**. Plots applied with PSB (+) had produced more numbers of grains ear^-1^ and grains row^-1^, heavy 1000 grains weight, higher grain yield and shelling percentage than plots without PSB (-). However, no significant differences were observed for harvest index between the plots with PSB (+) and without PSB (-). According to Hameeda et al. (2006) and Yazdani et al. (2009), inoculation of maize seed with PSB under greenhouse and field conditions increased yield and yield components. Increase in maize yield with PSB inoculation may be due to the promotion of root growth which in turn enhancing nutrients and water uptake from the soil (Lin et al., 1983). According to Balochgharayi (2011), application of beneficial microbes (*Pseudomonas*) significantly increased maize yield over control. Dobbelaere et al. (2002) assessed the inoculation effect of beneficial microbes on growth of spring wheat and observed that inoculated wheat plants had better growth, more number of grains spike^-1^ and grain yield.

#### Maize Response to Compost and PSB Interaction

**FIGURE 3:**
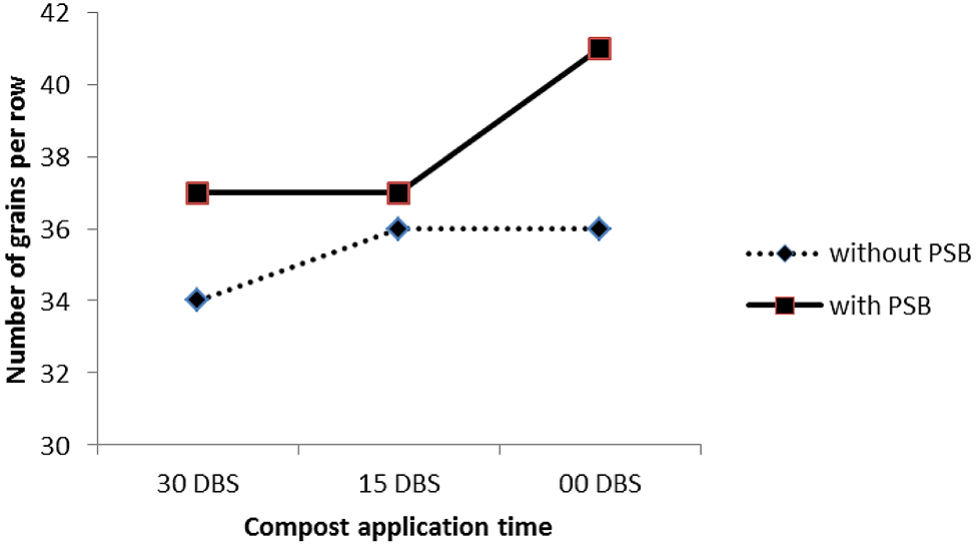
**Response of number of grains per row of maize (*Zea mays* L.) to compost application time (C) and phosphate solubilizing bacteria (PSB) interaction (C × PSB)**.

**FIGURE 4:**
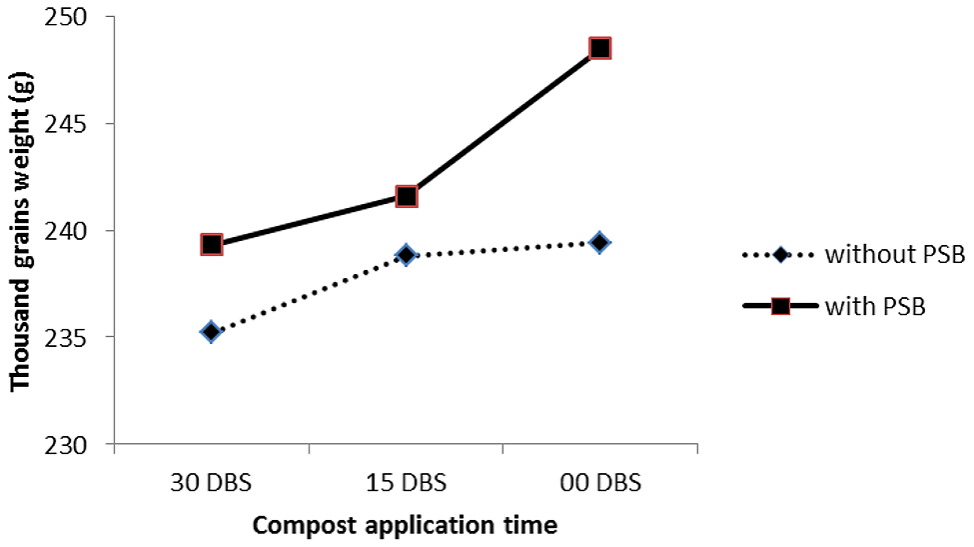
**Response of number of 1000 grains weight (g) of maize (*Zea mays* L.) to compost application time (C) and phosphate solubilizing bacteria (PSB) interaction (C × PSB)**.

**FIGURE 5:**
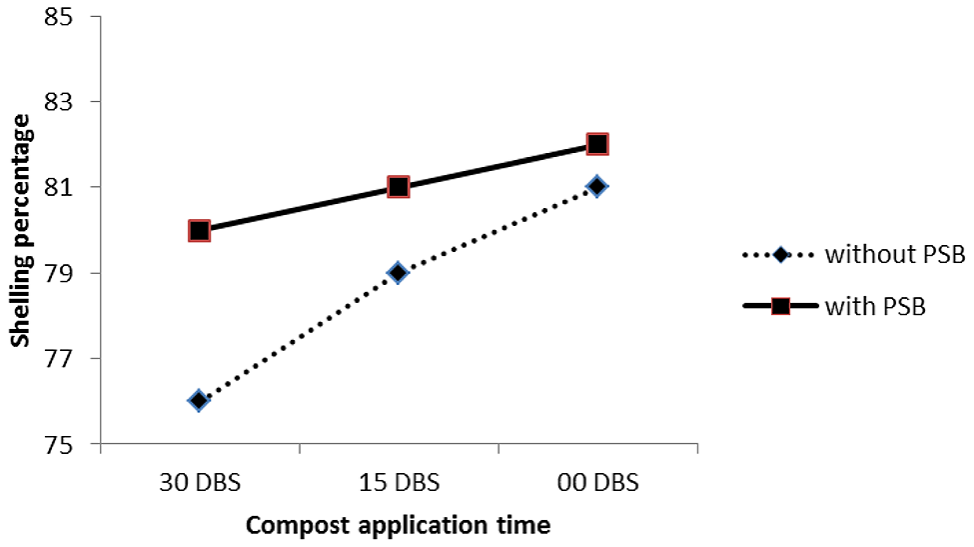
**Response of shelling percentage (%) of maize (*Zea mays* L.) to compost application time (C) and phosphate solubilizing bacteria (PSB) interaction (C × PSB)**.

The interaction between compost application time and PSB (with and without) (C × PSB) had significant effect on number of grains per row (**Figure 3**) and 1000 grains weight (**Figure 4**). The C × PSB (**Figure 3**) indicated that maize seed treat with PSB had produced more number of grains per row at all three timings of compost. However, the increase in grains per row ranked first in the PSB treated plots that received that compost at sowing time (**Figure 3**). The C × PSB (**Figure 4**) indicated that maize seed treat with PSB had produced heavier 1000 grains weight at all three timings of compost. The 1000 grains weight reached maximum level under the PSB treated plots that received that compost at sowing time (**Figure 4**). The C × PSB indicated that delay in compost application time had significantly increased the shelling percentage in maize gown in both with and without PSB treated plots and vice versa (**Figure 5**). The delay in compost application along with PSB probably may have increased P availability and uptake by maize plants thereby improved maize growth and produced higher yield and yield components. These results are in agreement with those of Singh and Amberger (1991) who reported that solubilization of phosphorus by organic acids released from compost increased P availability to plants. According to Cheuk et al. (2003) and Singh et al. (2004), compost application increased microbial activities and availability of mineral nutrients for crop plants. Becking (2006) reported that carbon content decreased in compost due to the increasing population of microorganisms which utilize decomposable organic waste both as a source of food and energy. Ravindrana et al. (2007) noticed reduction in pH, organic carbon and C:N ratio during incubation of farmyard manure and *phosphobacteria* compost.

#### Maize Response to Phosphorus and PSB Interaction

**FIGURE 6:**
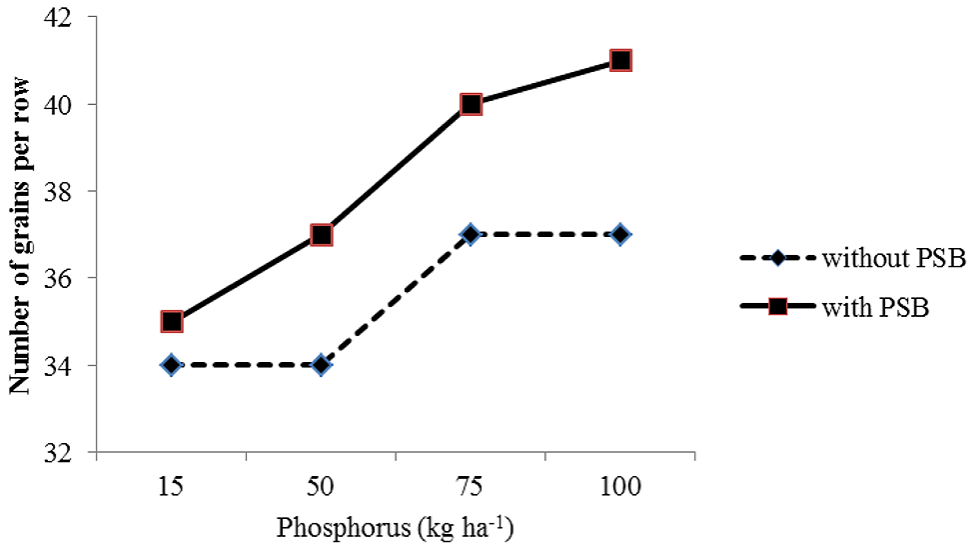
**Response of number of grains per row of maize (*Zea mays* L.) to phosphorus levels (P) and phosphate solubilizing bacteria (PSB) interaction (P × PSB)**.

**FIGURE 7:**
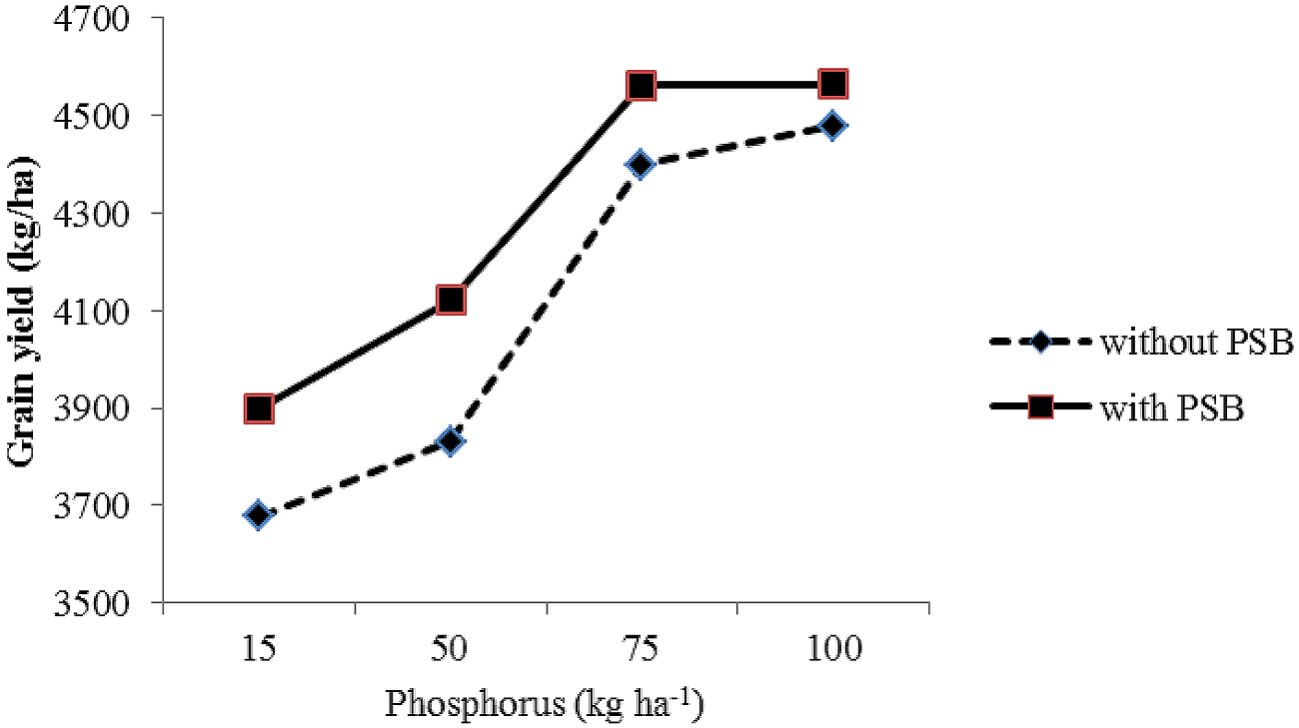
**Response of grain yield (kg/ha) of maize (*Zea mays* L.) to phosphorus levels (P) and phosphate solubilizing bacteria (PSB) interaction (P × PSB)**.

The interaction between P levels and PSB (P × PSB) had significant effect on number of grains per row (**Figure 6**) and grain yield (**Figure 7**). The P × PSB interaction indicated that the two higher P levels (75 and 100 kg P ha^-1^) had produced significantly more number of grains per row in maize grown under both with (+) and without PSB (-) treated plots (**Figure 6**). It was cleared from **Figure 6** that maize seed treatment with PSB (+) had produced more number of grains row of maize than the plots without PSB (-) under semiarid climates. The P × PSB interaction indicated the increase in P level increased grain yield in maize under both PSB treated (+) and PSB untreated (-) plots (**Figure 7**). The two higher P levels (75 and 100 kg P ha^-1^) had produced significantly higher grain yield grain yield than the two lower levels of P (25 and 50 Kg P ha^-1^) in the plots treated with (+) and plots where PSB was not applied (**Figure 7**). Interestingly, the combination of 75 Kg P ha^-1^ + PSB treated plots ranked first in terms of highest grain yield in the study area under semiarid climate. The higher rates of P along with PSB probably may have increased the availability and uptake of P from the soil that have positive impact on yield and yield components of maize. Banik and Dey (1981) reported that combined application of rock phosphate along with inoculation *Bacillus* and *Penicillinm* species increased availability of P. Many researchers (Kumar et al., 2001; Chen et al., 2006; Adesemoye and Kloepper, 2009) suggested that seed inoculation with PSB along with application pf soluble phosphatic fertilizer decrease P fixation on calcareous thereby increase P use efficiency and yield. Chaturvedi (2006) reported taller plants, more number of tillers m^-2^, leaf area, weight, leaf area index, total dry matter accumulation number of grains per spike, 1000 grain-weight, seed yield and N, P, and K uptake in wheat crop with application of P along with phosphate-solubilizing bacteria *(Pseudomonas striata*) and farmyard manure. Kushwaha (2007) and Patil et al. (2011) suggested that interaction among rock phosphate × PSB × compost increased P availability and crop yields.

## Conclusion

Application of phosphorus ate the rate of 75 kg P ha^-1^ was found most beneficial in terms of higher yield and yield components of maize under semiarid condition. Application of compost at sowing time produced higher yield and yield components of maize under semiarid condition. Maize seed inoculated with PSB had resulted in higher yield and yield components of maize over uninsulated seed with PSB.

## Conflict of Interest Statement

The authors declare that the research was conducted in the absence of any commercial or financial relationships that could be construed as a potential conflict of interest.
